# The lipidomic correlates of epigenetic aging across the adult lifespan: A population‐based study

**DOI:** 10.1111/acel.13934

**Published:** 2023-07-26

**Authors:** Dan Liu, N. Ahmad Aziz, Elvire Nadieh Landstra, Monique M. B. Breteler

**Affiliations:** ^1^ Population Health Sciences German Center for Neurodegenerative Diseases (DZNE) Bonn Germany; ^2^ Department of Neurology, Faculty of Medicine University of Bonn Bonn Germany; ^3^ Institute for Medical Biometry, Informatics and Epidemiology (IMBIE), Faculty of Medicine University of Bonn Bonn Germany

**Keywords:** biological aging, cohort studies, DNA methylation, epidemiology, lipidomics, odd‐numbered fatty acids

## Abstract

Lipid signaling is involved in longevity regulation, but which specific lipid molecular species affect human biological aging remains largely unknown. We investigated the relation between complex lipids and DNA methylation‐based metrics of biological aging among 4181 participants (mean age 55.1 years (range 30.0–95.0)) from the Rhineland Study, an ongoing population‐based cohort study in Bonn, Germany. The absolute concentration of 14 lipid classes, covering 964 molecular species and 267 fatty acid composites, was measured by Metabolon Complex Lipid Panel. DNA methylation‐based metrics of biological aging (AgeAccelPheno and AgeAccelGrim) were calculated based on published algorithms. Epigenome‐wide association analyses (EWAS) of biological aging‐associated lipids and pathway analysis were performed to gain biological insights into the mechanisms underlying the effects of lipidomics on biological aging. We found that higher levels of molecular species belonging to neutral lipids, phosphatidylethanolamines, phosphatidylinositols, and dihydroceramides were associated with faster biological aging, whereas higher levels of lysophosphatidylcholine, hexosylceramide, and lactosylceramide species were associated with slower biological aging. Ceramide, phosphatidylcholine, and lysophosphatidylethanolamine species with odd‐numbered fatty acid tail lengths were associated with slower biological aging, whereas those with even‐numbered chain lengths were associated with faster biological aging. EWAS combined with functional pathway analysis revealed several complex lipids associated with biological aging as important regulators of known longevity and aging‐related pathways.

## Graphical abstract

Leveraging high‐throughput lipidomic analyses covering 14 lipid classes, 964 molecular species, and 267 fatty acid composites in a population‐based study, we found that diverse complex lipid species were associated with different rates of biological aging. Both lipid class and fatty acid chain length and saturation were identified as key determinants of their influence on biological aging.

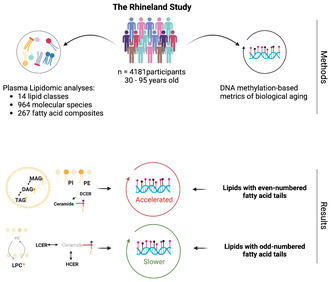

## 1 INTRODUCTION

The rapid aging of the global population has increased the personal and societal burden of age‐associated diseases and disabilities, warranting the urgent development of novel strategies for stimulating healthy aging (WHO, 2015). Individuals with the same chronological age often exhibit heterogeneous trajectories of age‐related functional decline as well as marked differences in their risk of morbidity and mortality (Beard et al., 2016; Levine, 2013; Lowsky et al., 2014). Understanding the drivers of different rates of biological aging could enable the development of more tailored and effective preventive and therapeutic strategies aimed at maximizing health span.

Dynamic DNA methylation regulates gene expression and is responsive to environmental and lifestyle changes. With increasing age, the methylation status of numerous DNA cytosine‐phosphate‐guanine (CpG) sites differentially changes across the genome (Bell et al., 2012, 2019; Florath et al., 2014; Horvath, 2013; Horvath & Raj, 2018; Jones et al., 2015). Hence, DNA methylation patterns have been used to estimate biological age through so‐called epigenetic clocks, also known as epigenetic aging estimators. The most recent of these include DNAm Phenotypic Age (PhenoAge), trained on mortality‐related clinical biomarkers (Levine et al., 2018), and DNAm GrimAge, developed using plasma proteins that are associated with age‐related conditions (Lu et al., 2019). Both PhenoAge and GrimAge more closely capture the high inter‐individual variability in the underlying biological aging processes and are strong predictors of mortality (Beynon et al., 2022; Hillary et al., 2021; Lemke et al., 2022; Li et al., 2021; Protsenko et al., 2021; Roberts et al., 2021; Verschoor et al., 2021). Recent studies have shown that GrimAge outperforms other biological age estimators in the prediction of age‐related diseases and all‐cause mortality and in reflecting multisystem dysfunction (Liu et al., 2023; McCrory et al., 2021; Protsenko et al., 2021).

The determinants of biological aging remain largely unknown, yet lipid metabolism has been suggested to be involved (Hahn et al., 2017; Mutlu et al., 2021). Key pathways that have been implicated in the aging process, including the insulin‐like growth factor‐Akt–mTOR pathway, the nuclear factor kB (NF‐kB) pathway, and the AMP‐activated protein kinase (AMPK) pathway, are also crucial regulators of lipid metabolism (Jesko et al., 2019; van der Spoel et al., 2015; Weir et al., 2017). Various circulating lipid species have been linked to age‐related phenotypes, including cardiovascular diseases (Tabassum et al., 2019), insulin resistance (Lemaitre et al., 2018), obesity (Yin et al., 2020), chronic kidney disease (Afshinnia et al., 2021), and Alzheimer's disease (Huynh et al., 2020). Further evidence for the crucial role of lipid metabolism in aging stems from studies of nonagenarians and centenarians, as well as their offspring, which found favorable lipid profiles in healthy agers (Collino et al., 2013; Gonzalez‐Covarrubias et al., 2013; Montoliu et al., 2014; Pradas et al., 2019; Vaarhorst et al., 2011). Notably, in the Leiden Longevity Study, it was found that the offspring of nonagenarians had higher levels of phosphocholine (PC) and sphingomyelin (SM) species and lower levels of phosphoethanolamine (PE) (38:6) and long‐chain triacylglycerols (TAGs), independent of total triglyceride levels (Gonzalez‐Covarrubias et al., 2013; Vaarhorst et al., 2011). Similarly, Pradas et al. found that higher levels of alkyl‐PC with shorter chain lengths and double bonds and/or lower levels of alkenyl‐PE with longer chain lengths and double bonds were associated with human longevity (Pradas et al., 2019).

Levels of blood lipids, including high‐density lipoprotein (HDL) and low‐density lipoprotein (LDL) cholesterol, TAGs, total cholesterol, and lipoprotein subfractions, have been associated with DNA methylation status (Braun et al., 2017; Dekkers et al., 2016; Frazier‐Wood et al., 2014; Gomez‐Alonso et al., 2021; Hedman et al., 2017; Irvin et al., 2014; Nuotio et al., 2020; Pfeiffer et al., 2015; Xie et al., 2019). A recent large‐scale epigenome‐wide association study (EWAS) (*N* = 16,265) found that hundreds of CpGs were associated with HDL, LDL, and TAGs in either trans‐ethnic or ethnic‐specific meta‐analyses (Jhun et al., 2021). EWAS combined with Mendelian randomization analyses indicated that inter‐individual variations in lipid levels are likely causally related to changes in DNA methylation (Dekkers et al., 2016; Jhun et al., 2021). Furthermore, many of the lipid‐related CpGs have also been linked to age‐related phenotypes, including metabolic syndrome, type 2 diabetes, and coronary artery disease (Gomez‐Alonso et al., 2021; Hedman et al., 2017; Xie et al., 2019). Taken together, current evidence suggests that variations in lipid levels may exert at least part of their (pathological) effects through epigenomic remodeling.

Despite the intriguing connection between lipid metabolism and aging, it is still unknown whether and how inter‐individual differences in lipid profiles contribute to different rates of biological aging in the general population. The heterogeneous chemical structure of lipids poses challenges for their accurate quantification, and until now only a few lipid species have been investigated in the context of human aging and age‐related health outcomes. Yet the vast diversity of lipid functions is reflected by the wide variation in the structure and composition of lipid molecules, which ultimately determine their specific effects (Harayama & Riezman, 2018). Recently, high‐throughput, in‐depth molecular characterization of many lipid species has become available through Metabolon's complex lipid assay platform (Wu et al., 2022). This lipidomics platform provides absolute quantitation of 14 lipid classes across phospholipids, sphingolipids, and neutral lipids, as well as the complete fatty acid composition of each lipid class, covering more than 900 absolute concentrations of their constituent molecular species. These recent technological developments make it possible to investigate the contribution of complex lipids to biological aging at a population level. Furthermore, we performed epigenome‐wide association analyses of biological aging‐associated lipid molecular species and pathway analysis to gain biological insights into the mechanisms underlying the effects of lipidomics on biological aging.

## 2 RESULTS

### 2.1 Estimations of AgeAccelPheno and AgeAccelGrim

The characteristics of the study population are presented in Table 1. AgeAccelPheno and AgeAccelGrim were significantly higher in men than in women. The concentrations of the measured plasma lipid classes are consistent with the quantitative plasma lipidomic analyses of a similar study (Eichelmann et al., 2022).

**TABLE 1:** Characteristics of the study population.

	Overall (*N* = 4181)	Women (*N* = 2354)	Men (*N* = 1827)	Adjusted *p*‐value*
Age, year				
Mean (SD)	55.1 (14.0)	54.9 (13.7)	55.3 (14.4)	<0.001
Median [Min, Max]	55.0 [30.0, 95.0]	54.0 [30.0, 95.0]	55.0 [30.0, 91.0]	
Current smoking, *n* (%)	529 (12.7%)	279 (11.9%)	250 (13.7%)	0.068
BMI, kg/m^2^, mean (SD)	25.9 (4.5)	25.4 (4.8)	26.5 (3.9)	<0.001
SBP, mmHg, mean (SD)	126 (15.9)	123 (16.4)	131 (14.1)	<0.001
DBP, mmHg, mean (SD)	75.5 (9.4)	73.8 (9.1)	77.5 (9.3)	<0.001
Hypertension, *n* (%)	1569 (37.5%)	783(33.2%)	786 (43.1%)	<0.001
Diabetes, *n* (%)	218 (5.2)	85 (3.6%)	133 (7.3%)	<0.001
HDL, mg/dL	62.6 (17.9)	69.9 (17.3)	53.2 (13.9)	<0.001
LDL, mg/dL	126 (35.6)	126 (36.4)	127 (34.6)	0.137
Cholesterol, mg/dL	199 (39.3)	203 (39.6)	194 (38.4)	<0.001
Triglyceride, mg/dL	112 (70.4)	97.9 (51.7)	130 (85.6)	<0.001
DNAm age acceleration estimators, year, mean (SD)				
AgeAccelPheno	0.2 (6.6)	−0.3 (6.7)	0.8 (6.4)	<0.001
AgeAccelGrim	0.0 (6.8)	−1.0 (6.6)	1.3 (6.8)	<0.001
Complex lipid class, μM, mean (SD)				
Neutral lipids				
Cholesteryl esters (CE)	2870 (645)	2900 (654)	2820 (632)	<0.001
Monoacylglycerol (MAG)	2.2 (5.34)	2.1 (3.99)	2.4 (6.69)	0.070
Diacylglycerols (DAG)	25.9 (16.2)	22.8 (12.1)	29.9 (19.6)	<0.001
Triacylglycerols (TAG)	1060 (667)	924 (511)	1230 (794)	<0.001
Phospholipids				
Phosphatidylcholine (PC)	2010 (408)	2080 (401)	1920 (399)	<0.001
Phosphatidylethanolamine (PE)	157 (42.8)	161 (41.6)	152 (43.7)	<0.001
Phosphatidylinositol (PI)	38.1 (10.2)	39.2 (10.2)	36.8 (10.1)	<0.001
Lysophosphatidylcholine (LPC)	163 (38.4)	156 (36.6)	171 (38.9)	<0.001
Lysophosphatidylethanolamine (LPE)	6.2 (2.1)	6.1 (2.1)	6.3 (2.2)	0.001
Sphingolipids				
Sphingomyelin (SM)	486 (89.3)	505 (90.0)	460 (81.9)	<0.001
Ceramide (CER)	5.1 (1.4)	5.0 (1.4)	5.2 (1.4)	<0.001
Dihydroceramide (DCER)	1.3 (0.4)	1.3 (0.4)	1.3 (0.5)	<0.001
Hexosylceramide (HCER)	4.3 (1.2)	4.4 (1.2)	4.3 (1.1)	0.077
Lactosylceramide (LCER)	3.4 (0.8)	3.5 (0.8)	3.3 (0.7)	<0.001

*Note*: The missingness for each variable is less than 5%.

Abbreviations: BMI, body mass index; DBP, diastolic blood pressure; HDL, high‐density lipoproteins; LDL, low‐density lipoproteins; SBP, systolic blood pressure; SD, standard deviation.

* Comparison between women and men, adjusted for age.

### 2.2 Associations of lipid class and molecular species with AgeAccelPheno and AgeAccelGrim

Age, sex, and batch‐adjusted partial correlations showed that all lipid classes except for MAG, LPE, and LPC were moderately to highly correlated (Pearson's *r* > 0.3). The strongest correlations for LDL and cholesterol were with CE, PC, PI, and sphingolipids, whereases HDL was only weakly correlated with almost all main lipid classes. As expected, clinically measured total triglycerides were highly correlated with DAG and TAG, and moderately correlated with phospholipids and sphingolipids. BMI was only weakly correlated with the main lipid classes (Figure S1).

**FIGURE 1:**
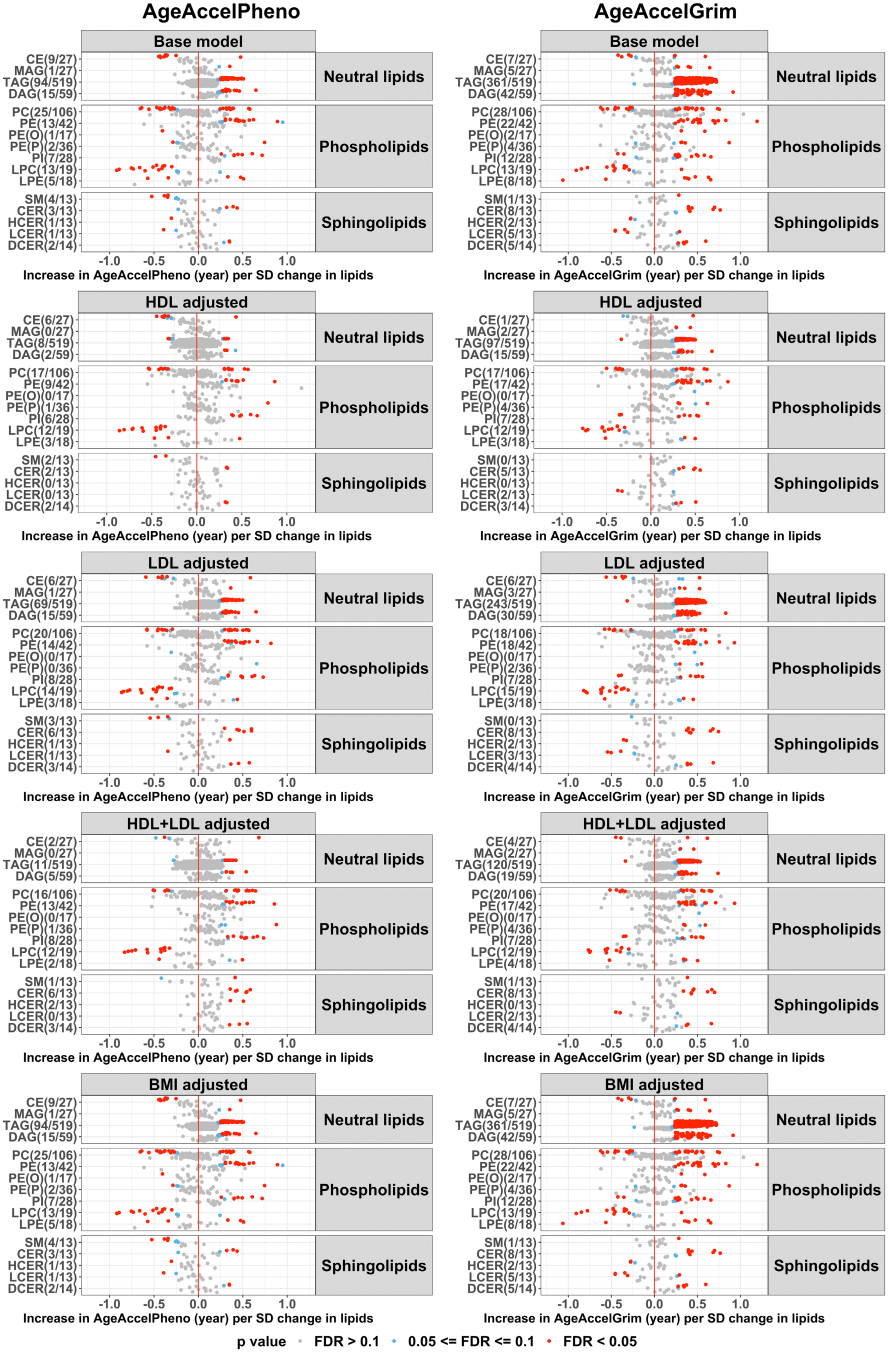
Associations of lipid species with AgeAccelPheno and AgeAccelGrim. Each dot represents one molecular species; the dot color indicates the significance level. Base model: AgeAccel ~ lipid + sex + batch information + smoking status. BMI, body mass index; CE, cholesteryl ester; CER, ceramide; DAG, diacylglycerol; DCER, dihydroceramide; FDR, false discovery rate; HCER, hexosylceramide; HDL, high‐density lipoprotein; LCER, lactosylceramide; LDL, low‐density lipoprotein; LPC, lysophosphatidylcholine; LPE, lysophosphatidylethanolamine; MAG, monoacylglycerol; PC, phosphatidylcholine; PE(O), phosphatidylethanolamine ether; PE(P), phosphatidylethanolamine plasmalogen; PE, phosphatidylethanolamine; PI, phosphatidylinositol; SM, sphingomyelin; TAG, triacylglycerol.

Out of 964 lipid species, 196 and 525 were associated with AgeAccelPheno and AgeAccelGrim at FDR < 0.05, respectively (Figure 1). For neutral lipids (MAG, TAG, DAG), phospholipids (PE, PI), and sphingolipids (CER, DCER), per SD concentration increase in each molecular species, AgeAccelPheno and AgeAccelGrim increased with around 0.25–0.75 year. For LPC, HCER, and LCER classes, AgeAccelPheno and AgeAccelGrim decreased with 0.25–1.00 year per each SD concentration increase in each molecular species. More molecular species were associated with AgeAccelGrim, and with larger effect sizes, than with AgeAccelPheno (Figure 1).

After adjustment for HDL and/or LDL levels, the associations of many molecular species belonging to the neutral lipids and phospholipids classes—especially TAG, DAG, PC, and PE—with AgeAccelPheno and AgeAccelGrim became non‐significant, Conversely, adjustment for HDL and/or LDL levels did not materially affect the results for sphingolipids. These findings are in line with the known function of lipoproteins as key regulators of mainly neutral lipids and phospholipids metabolism, whereas they have a smaller influence on sphingolipids metabolism (Borodzicz et al., 2015). The results remained almost identical after adjustment for BMI (Figure 1).

We found that DAG, MAG, and TAG levels tended to be stable across the different age groups and PI levels tended to decrease over the years. Lipid levels in CE, PC, and sphingolipids tended to decrease from the age of 60, with significantly lower levels of these lipids in participants from the 80–89 age group. Unfortunately, only seven participants were older than 90 years, which rendered it impossible to obtain precise estimates for this age group as reflected in the vary wide confidence intervals (Figure S2).

### 2.3 Associations of the total number of carbons and double bonds with AgeAccelPheno and AgeAccelGrim

**FIGURE 2:**
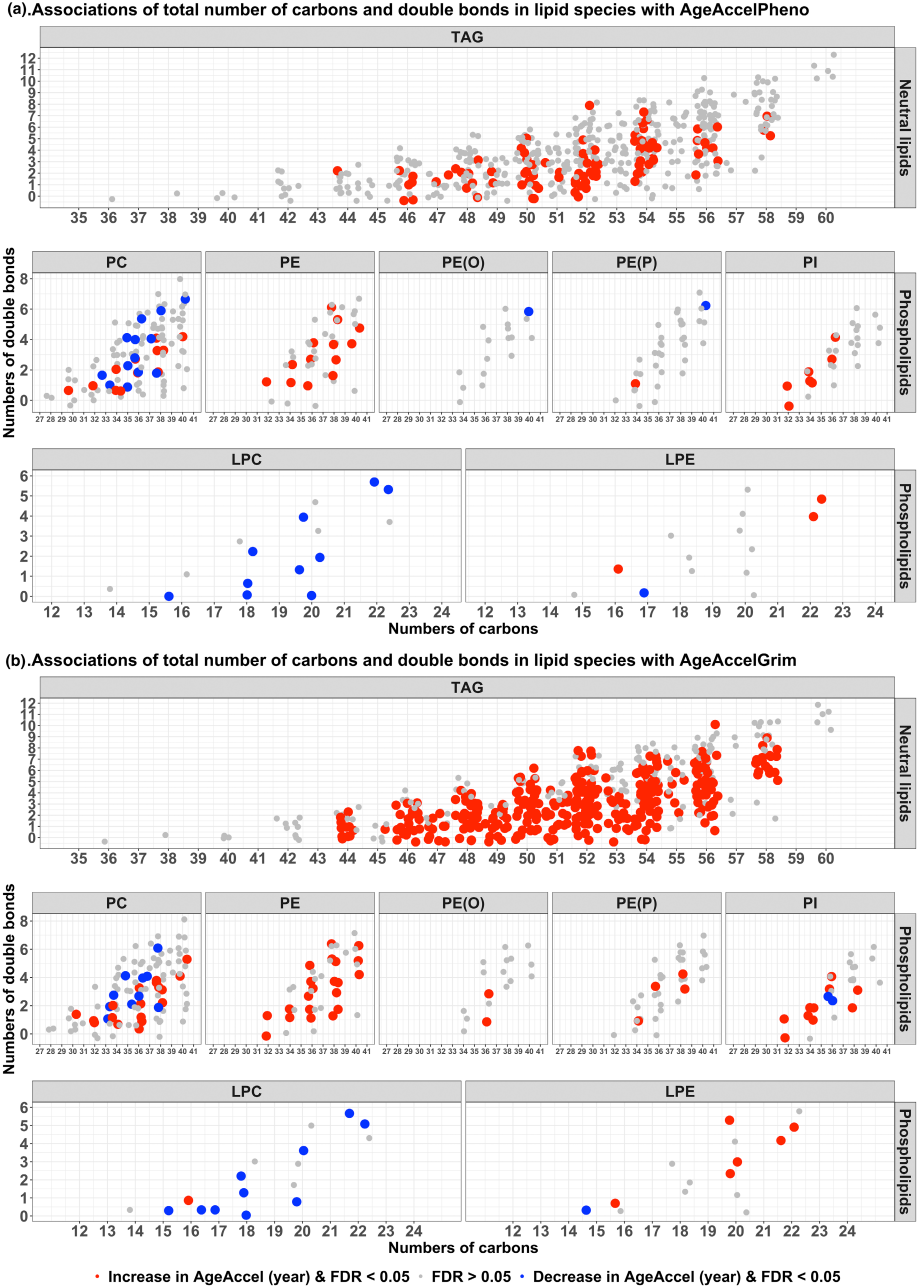
Associations of the total number of carbons and double bonds in lipid species with AgeAccelPheno and AgeAccelGrim. Individual lipid species are depicted by filled circles and arranged by lipid class according to the total number of carbon atoms (x‐axes) and the number of double bonds (y‐axes). The circle color indicates the magnitude and direction (positive or negative) of the effect size, and the circle size corresponds to the significance level. Lipids with the same number of carbon atoms and double bonds are pulled apart vertically to increase their visibility. FDR, false discovery rate; LPC, lysophosphatidylcholine; LPE, lysophosphatidylethanolamine; PC, phosphatidylcholine; PE(O), phosphatidylethanolamine ether; PE(P), phosphatidylethanolamine plasmalogen; PE, phosphatidylethanolamine; PI, phosphatidylinositol; TAG, triacylglycerol.

Within the neutral lipid category, higher levels of TAG molecular species with even numbers of carbons were associated with higher AgeAccelPheno (Figure 2a) and AgeAccelGrim (Figure 2b), whereas fewer significant associations were observed for species containing an odd‐numbered chain length. Regarding phospholipids, higher levels of molecular species belonging to PC, PE(O), PE(P), and PI classes with more double bonds (i.e., polyunsaturated fatty acids) and more carbons tended to be associated with lower AgeAccelPheno and AgeAccelGrim, whereas species with fewer double bonds and fewer carbons tended to be associated with higher AgeAccelPheno and AgeAccelGrim. Predominantly LPC and LPE molecular species with fewer carbons were associated with lower AgeAccelPheno and AgeAccelGrim. There was no clear pattern for the associations of other neutral lipids and sphingolipids species with AgeAccelPheno and AgeAccelGrim (Figure S3).

### 2.4 Associations of fatty acid composition across lipid classes with AgeAccelPheno and AgeAccelGrim

**FIGURE 3:**
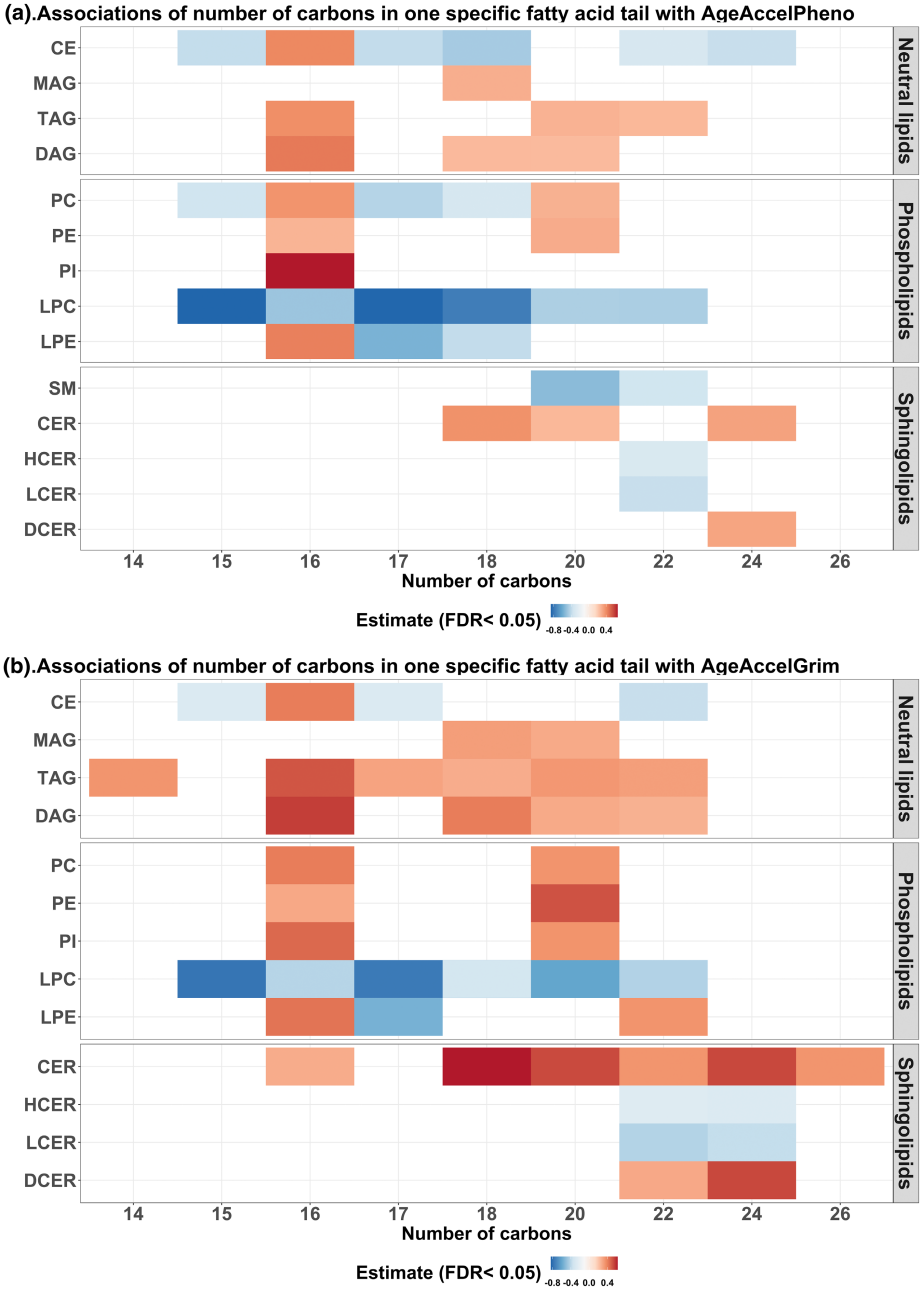
Associations of fatty acid compositions with AgeAccelPheno and AgeAccelGrim. The color indicates the magnitude of the effect size. To better visualize the results, only the effect size of lipid species at FDR <0.05 level was shown. CE, cholesteryl ester; CER, ceramide; DAG, diacylglycerol; DCER, dihydroceramide; HCER, hexosylceramide; LCER, lactosylceramide; LPC, lysophosphatidylcholine; LPE, lysophosphatidylethanolamine; MAG, monoacylglycerol; PC, phosphatidylcholine; PE, phosphatidylethanolamine; PI, phosphatidylinositol; SM, sphingomyelin; TAG, triacylglycerol.

The number of acyl chain carbons in a lipid's fatty acid tail may define specific biological effects (Harayama & Riezman, 2018). We analyzed 267 lipids classified based on one specific fatty acid tail, referred to as fatty acid composition, covering 14 lipid classes. Across lipid classes, the direction and strength of the effect on AgeAccelPheno and AgeAccelGrim were determined by the chain length of the fatty acid tail (Figure 3). Lipid molecular species with an even number (i.e., 14, 16, 18, 20, 22, 24, and 26) of carbons in the fatty acid tail were associated with higher AgeAccelPheno and AgeAccelGrim across lipid classes (except for LPC, HCER, and LCER), while lipid species with an odd number (i.e., 15 and 17) of carbons in the fatty acid tail were associated with lower AgeAccelPheno and AgeAccelGrim. In addition, shorter fatty acid tails were related to larger effect sizes. Importantly, the direction of the effects also depended on the lipid class (Figures 1 and 3).

### 2.5 Associations of saturation of fatty acid tails with AgeAccelPheno and AgeAccelGrim

**FIGURE 4:**
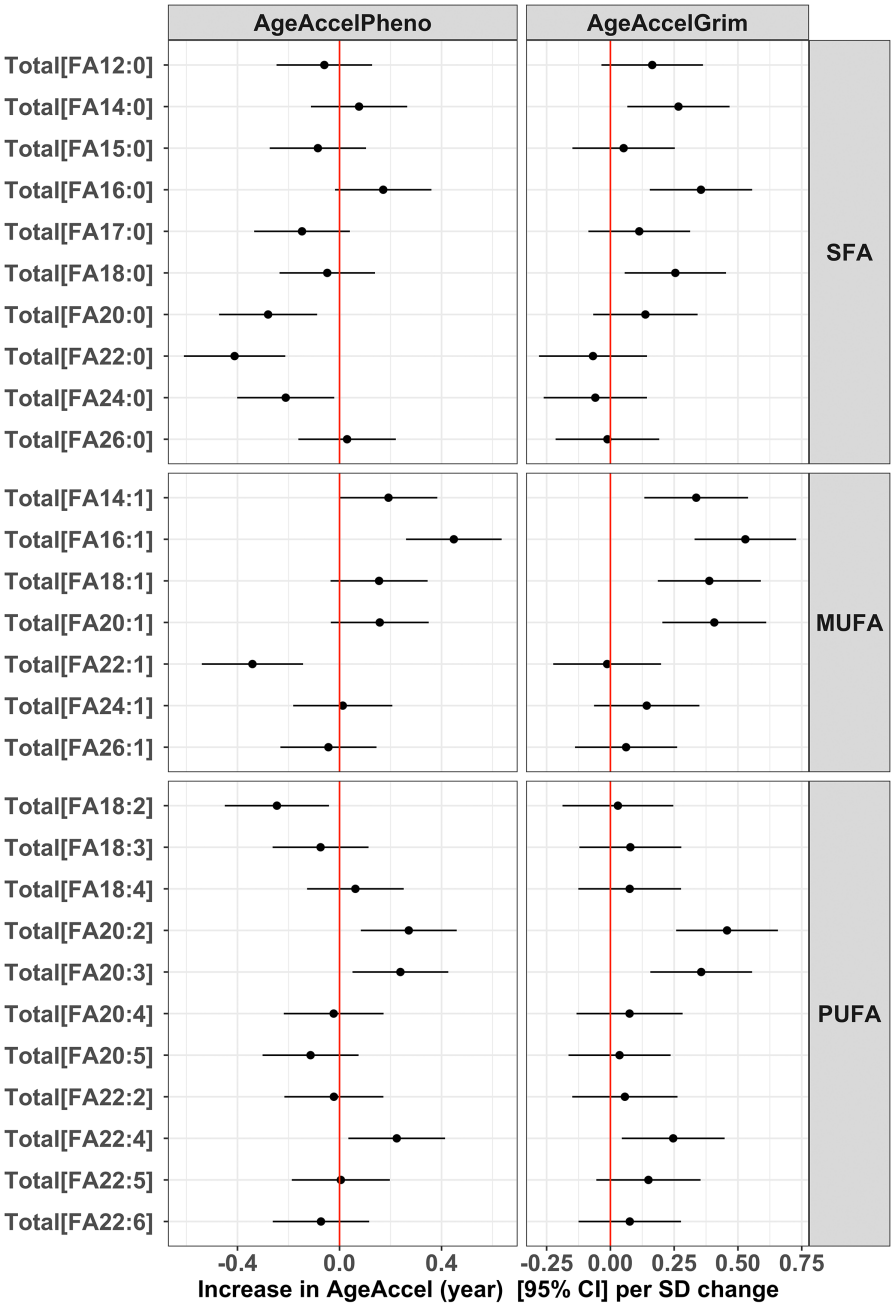
Associations of saturation of fatty acid tails with AgeAccelPheno and AgeAccelGrim. MUFA, monounsaturated fatty acids; PUFA, polyunsaturated fatty acid; SFA, saturated fatty acid.

Differences in the content and fraction of mono‐ and polyunsaturated lipids determine membrane peroxidation, which has been linked to longevity (Gonzalez‐Covarrubias et al., 2013; Pradas et al., 2019). For MUFA lipids, the effect on AgeAccelPheno and AgeAccelGrim was stronger with shorter (even numbered) chain lengths of the fatty acid tail. In contrast, for PUFA lipids with the same chain length, fewer double bonds were related to a stronger effect on AgeAccelPheno and AgeAccelGrim (Figure 4).

### 2.6 Sex interaction and sex‐stratified analyses

We found that lipid species, which were differently associated with AgeAccelPheno and AgeAccelGrim in men and women, predominately belonged to TAG and some phospholipid species (Figure S4), which is in line with reports from previous studies (Gonzalez‐Covarrubias et al., 2013; Hoene et al., 2014; Tabassum et al., 2022). Sex‐stratified analyses on these lipid species showed that each SD increase of the concentration of 2 MAG, 4 DAG or 51 TAG species, AgeAccelPheno increased with 0.5‐years in women, but not in men (Figure S5). Per SD increase of the concentration of PE(P‐16:0/16:0), MAG (18:1), MAG (18:2), and 8 DAG species, AgeAccelGrim increased with more than 0.5 years increase in women, whereases per SD increase of the concentration of PI (18:0/16:1) and PI (16:0/16:1), AgeAccelGrim increased with around 0.5 years in men. Higher concentrations of TAG (42:2‐FA18:2), TAG (42:2‐FA12:0), and TAG (40:0‐FA14:0) were only associated with lower AgeAccelGrim in women (Figure S6).

### 2.7 Uncovering biological pathways involved in the association of lipidome with AgeAccelPheno and AgeAccelGrim

**FIGURE 5:**
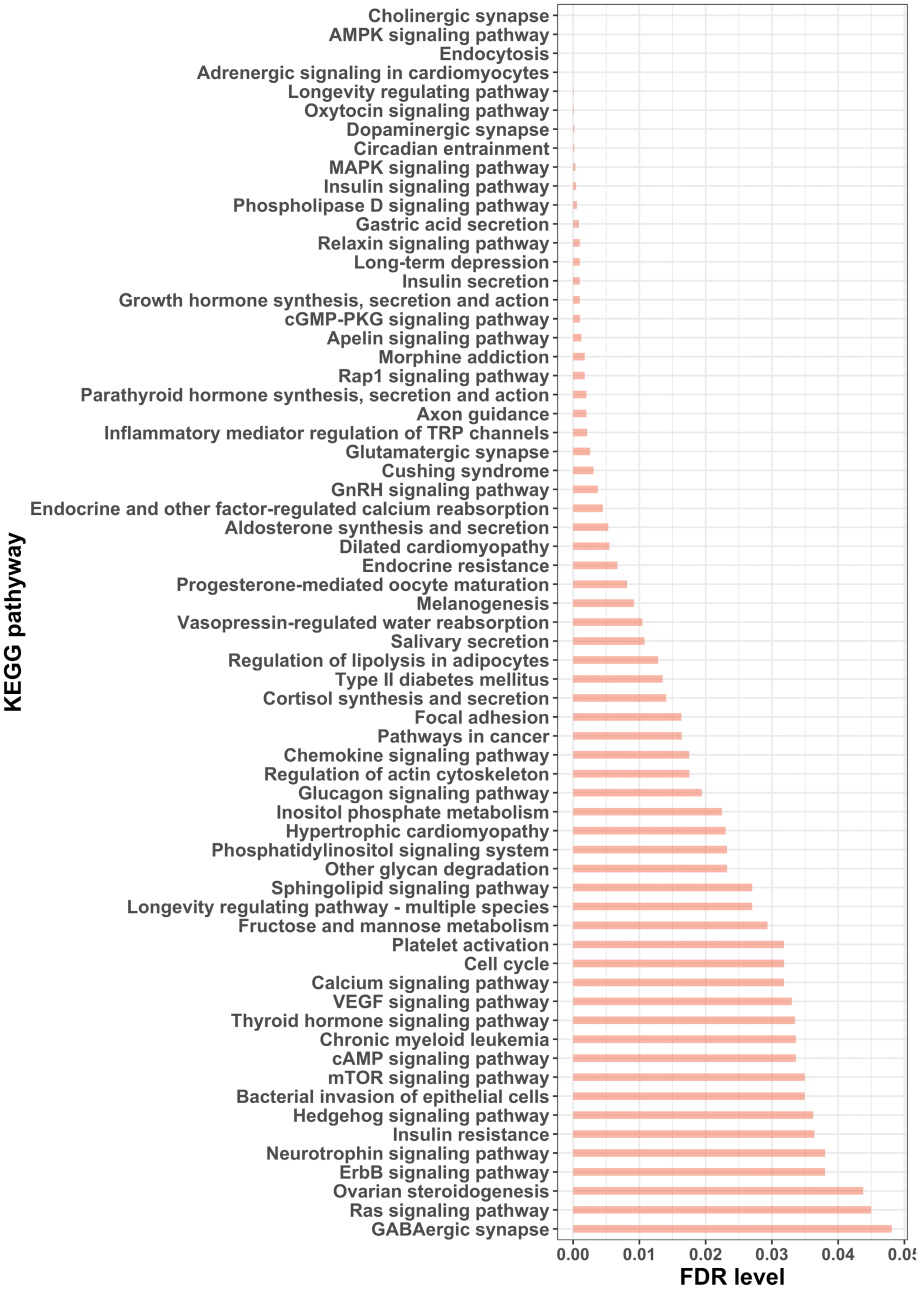
Biological pathways involved in the association of lipidome with AgeAccelPheno and AgeAccelGrim. FDR, false discovery rate.

Epigenome‐wide association analyses were performed for 525 lipids, which were identified as being associated with either AgeAccelPheno or AgeAccelGrim (Figure 1). This approach resulted in the identification of lipid‐associated CpGs that were subsequently used as uniquely defined proxies for the respective lipids, enabling KEGG pathway analysis to delineate the underlying biological pathways modulated by AgeAccel‐associated lipids. A total of 65 pathways were identified, including many known longevity‐related pathways such as the mTOR signaling pathway, AMPK signaling pathway, MAPK signaling pathway, and growth hormone synthesis, secretion, and action pathway. Moreover, pathways involved in age‐related diseases, including type 2 diabetes mellitus, insulin resistance and secretion, cortisol synthesis, and secretion, and long‐term depression were among those related to lipid‐associated CpGs. Importantly, molecular pathways associated with brain function were highlighted, including cholinergic synapse, dopaminergic synapse, axon guidance, neurotrophy signaling pathway, and GABAergic synapse (Figure 5).

## 3 DISCUSSION

We investigated 14 complex lipid classes, covering 964 molecular species and 267 fatty acid composites, with biological aging. We found complex lipid species to be differently associated with different rates of biological aging. Higher levels of molecular species belonging to the neutral lipids (MAG, DAG, TAG), phospholipids (PE, PE(O), PE(P)), and sphingolipids (CER, DCER) classes were associated with accelerated biological aging, whereas higher levels of distinct other molecular species (i.e., LPC, HCER, and LCER) were associated with slower biological aging. CE, PC, and LPE molecular species with odd‐numbered (i.e., 15 and 17) fatty acid tail lengths were associated with slower biological aging, yet even‐numbered fatty acid tail lengths were associated with faster biological aging. Importantly, in silico pathway analysis revealed that lipids that were associated with biological aging estimators were mainly involved in known longevity and aging‐related pathways, revealing their role as potential determinants of biological aging across the lifespan in the general population.

Our observation that higher levels of odd‐numbered fatty acid tail lengths (15:0 and 17:0) were associated with slower biological aging, whereas even‐numbered fatty acid tail lengths were associated with faster biological aging, fits with findings from previous studies. The EPIC‐InterAct study (*n* = 27,296) found that higher levels of odd‐chain saturated fatty acids (15:0; 17:0) were associated with a reduced risk of type 2 diabetes, whereas the risk was increased for people with higher levels of even‐chain saturated fatty acids (Forouhi et al., 2014). Likewise, the EPIC‐Norfolk study (*n* = 7354) found that higher levels of even‐chain saturated fatty acids were associated with a higher risk of incident coronary heart disease (CHD), while odd‐chain saturated fatty acids (15:0; 17:0) were associated with a lower risk of incident CHD (Khaw et al., 2012). However, the underlying mechanisms are largely unknown. Previous studies have suggested that circulating C15:0 could be regarded as a direct biomarker of dietary C15:0 intake (Gotoh et al., 2008; Hodson et al., 2008; Jenkins et al., 2017; Rezanka & Sigler, 2009; Santaren et al., 2019), whereas circulating C17:0 is a product of biosynthesis, which can be affected by and correlates with dietary substrate intake (Jenkins et al., 2017). Even‐numbered chain‐length saturated fatty acids are mainly derived from de novo lipogenesis, through which carbohydrates and alcohol are converted to fatty acids in the liver or adipose tissue. Biochemical experiments also demonstrated toxic effects of 16:0, 18:0, and 24:0, including activation of inflammatory cytokines and lipotoxicity to pancreatic β cells (Jalili & Hekmatdoost, 2021; Zhou et al., 2020). This suggests that the lipid composition of the diet might have an impact on the rate of biological aging.

Very little work has explicitly assessed the value of LPC species as potential human blood‐derived biomarkers of human aging. Circulating LPCs are generated by phospholipases A2 from the PC. The most abundant LPC in human plasma is 16:0, followed by 18:2, 18:0, 18:1, 20:4, and other minor species (Drzazga et al., 2014). Here we found that higher levels of 13 out of 19 LPC species exhibit a robust association with slower biological aging, suggesting that LPC species may contribute to healthy aging. Our findings expand on those from recent epidemiological studies, which assessed a limited number of LPC species, and reported low concentrations of certain circulating LPCs (i.e., 18:2 and/or 17:0) to be associated with several aging‐related phenotypes and disorders, including memory impairment (Mapstone et al., 2014), gait speed decline (Gonzalez‐Freire et al., 2019), and incident myocardial infarction (Ward‐Caviness et al., 2017). Moreover, elevated LPC (18:1) levels have been reported in centenarians (Montoliu et al., 2014). Potential biological mechanisms through which LPCs could contribute to slower biological aging and less age‐associated functional decline are antioxidative stress and anti‐inflammatory responses (Knuplez & Marsche, 2020; Law et al., 2019). Polyunsaturated LPCs (e.g., LPC 20:4, 22:6) were shown to exert anti‐inflammatory effects in animal studies (Hung et al., 2012). Recently, lower levels of several LPC species (LPC 16:0, 16:1, 17:0, 18:1, 18:2, 20:3) were also linked to impaired mitochondrial oxidative capacity in adults, another important hallmark of aging (Semba et al., 2019).

The major phospholipids in eukaryotic biomembranes are phosphatidylcholine (PC), and phosphatidylethanolamine (PE) (van Meer et al., 2008), which were also quantified in our study. PC can be synthesized by a three‐step methylation of PE (Shields et al., 2001; Vance & Ridgway, 1988). We found that higher levels of various PE species were related to accelerated biological aging across the lifespan, whereas higher levels of polyunsaturated PCs were associated with slower biological aging. Higher levels of species with fewer double bonds tended to be associated with accelerated biological aging. These findings are in line with previous studies that found associations between higher levels of saturated and monounsaturated PCs and increased risk of cardiovascular diseases and type 2 diabetes (Tabassum et al., 2019). Conversely, polyunsaturated PC species have been linked to longevity, which might be due to their antioxidative and cardioprotective properties (Gonzalez‐Covarrubias et al., 2013; Montoliu et al., 2014; Pradas et al., 2019). PE species, the second most abundant membrane phospholipids, have been identified as modulators of inflammation and apoptosis (Tian et al., 2020), yet little is known about the properties of specific PE species. Here we show that higher levels of various PE species were related to accelerated biological aging across lifespan, thereby supporting and substantially extending previous reports of lower PE (38:6) levels in the offspring of nonagenarians (Gonzalez‐Covarrubias et al., 2013). Importantly, recent studies have shown that deficiency in PE methylation to synthesize PC leads to the elevation of S‐adenosylmethionine (SAM) levels, a major methyl donor. The SAM accumulation results in aberrant increases in nucleic acid methylation (i.e., histone methylation and DNA methylation), which subsequently leads to defects in the regulation of gene expression (Gao et al., 2018; Mahmoud & Ali, 2019; Ye et al., 2017). Our EWAS analyses identified numerous lipid‐associated CpGs, suggesting that changes in lipids levels were associated with DNA methylation reactions. Taken together, disruption in PE and PC biosynthesis could lead to SAM accumulation, which in turn alters the methylation reactions of nucleic acids, resulting in abnormal gene expression regulation, thereby contributing to biological aging.

Higher TAG levels are linked to an increased risk of cardiovascular diseases and Alzheimer's disease (Huynh et al., 2020; Tabassum et al., 2019). Small‐scale lipidomic profiling in longevity studies also found lower levels of TAG species (including TAG 46:5, 47:5, 52:1, 54:7, 54:6, 56:6, 56:7, 57:2) to be associated with healthy aging (Collino et al., 2013; Gonzalez‐Covarrubias et al., 2013; Montoliu et al., 2014). Our findings extend these previous reports by showing that 361 out of 519 TAG species across different chain lengths and double bonds were associated with accelerated biological aging. Few studies have investigated the association between other neutral lipids (including CE, MAG, and DAG) and longevity or healthy aging. We found that higher levels of DAG species or lower levels of CE species were related to an accelerated rate of biological aging, indicating that almost all neutral lipids could potentially influence longevity.

Distinct patterns of plasma sphingolipids have been linked to healthy aging and various age‐related diseases, including Alzheimer's disease (Huynh et al., 2020), Parkinson's disease (Avisar et al., 2021), diabetes (Fretts et al., 2021), and CVDs (Choi et al., 2021; Diaz et al., 2021). We found that higher levels of CER and DCER molecular species were associated with accelerated biological aging, whereas higher levels of SM, HCER, and LCER molecular species were associated with slower biological aging, which is in line with previous studies. Both genetic and observational studies have reported that higher levels of ceramides were associated with an increased risk of CVD events and diabetes (Diaz et al., 2021; Fretts et al., 2021; Tabassum et al., 2019), potentially through activation of NADPH oxidase and disruption of mitochondrial function (Choi et al., 2021). On the other hand, the offspring of long‐lived individuals were found to have higher levels of SM species (Gonzalez‐Covarrubias et al., 2013; Vaarhorst et al., 2011), while HCER species were increased in centenarians (Pradas et al., 2022). Moreover, lower levels of these healthy aging‐related SM species have previously been associated with diabetes and hypertension (Choi et al., 2021; Gonzalez‐Covarrubias et al., 2013; Vaarhorst et al., 2011).

Interestingly, the biological aging‐related lipidomic signatures substantially overlap with previously reported lipidomic profiles of neurodegeneration. Using two clinical cohort studies of Alzheimer's disease (sample size *n* = 1912), Huynh et al. (2020) identified hundreds of AD‐related lipid signatures. These lipid species were predominately from sphingolipids (CE, SM, HCER, and DECR), TAG, PE, and PC classes (Bernath et al., 2020; Huynh et al., 2020; Liu et al., 2021), which were also associated with biological aging in our study. For instance, higher levels of TAG species [TAG(52:2) and TAG(52:3)] and PE species (PE(P40:6) and PE(O40:6)) that previously were found to be associated with a higher risk of AD, were also related to accelerated biological aging in our study. Likewise, studies of non‐demented individuals reported that lipid species from TAG, CE, SM, PE, and PC classes were associated with cognitive decline and brain aging parameters (Bernath et al., 2020; Lefevre‐Arbogast et al., 2021; McGrath et al., 2020). Aging is the strongest risk factor for neurodegenerative diseases and accumulating evidence indicates that biological aging and neurodegeneration are interconnected (Xia et al., 2018). Indeed, our pathway analyses revealed that biological aging‐related lipids were enriched in molecular pathways associated with brain function, including cholinergic/dopaminergic/GABAergic synapse, axon guidance, and neurotrophy signaling pathways. Our findings thus indicate that biological aging processes driven by lipidomics alterations may, at least partly, contribute to brain aging and neurodegeneration.

Potential biological mechanisms through which changes in complex lipids levels could contribute to physiological and (neuro)pathological aging may include dysregulation of signal transduction via lipid rafts. Lipid rafts (LRs) are microdomains in the plasma membrane enriched in sphingolipids, cholesterol, and scaffolding proteins, which serve as a platform for signal transduction, cytoskeletal organization, and vesicular trafficking (Simons & Toomre, 2000). Previous studies have shown that LRs and their components regulate nutrient‐sensing pathways, calcium homeostasis, synaptic and neurotrophin signaling (Zhang et al., 2022). Indeed, our EWAS combined with functional pathways analyses revealed that biological aging‐related lipids converged on pathways related to lipid signaling pathways (i.e., sphingolipids, phosphatidylinositol and phospholipase D signaling pathway), nutrient‐sensing pathways (i.e., AMPK, MAPK, insulin, cAMP, and mTOR signaling pathway), and neuroplasticity pathways (i.e., cholinergic/glutamatergic/GABAergic synapse, axon guidance, calcium and neurotrophin signaling pathway). It is noteworthy that nutrient‐sensing alterations reportedly affect the lifespan (de Lucia et al., 2020). It is thus possible that plasma complex lipids fluctuations may disrupt LRs in lipids and nutrient‐sensing signaling, thereby contributing to biological aging (de Lucia et al., 2020; Zhang et al., 2022). In addition, LRs have been detected at synapses and are essential for synapse development, Aβ production, and cholesterol efflux. Complex lipid changes and subsequent loss of these microdomains may therefore possibly impact pre‐ and postsynaptic function, ultimately leading to (neuro)pathological aging (Egawa et al., 2016; Mesa‐Herrera et al., 2019).

HDL and LDL levels are well‐established biomarkers for various CVDs and are widely used in assessing CVD risk in the clinic (Singh et al., 2020; Vallejo‐Vaz et al., 2017). We demonstrate that the effects of neutral lipids and phospholipids on biological aging largely depend on HDL and LDL levels, whereas those of sphingolipids are largely independent of HDL and LDL levels. This finding reflects the well‐characterized role of lipoproteins in lipid metabolism: Lipoproteins are complex aggregates of lipids and proteins that render the hydrophobic lipids compatible with the body fluids and enable their transport throughout the body to tissues where they are required. Their most abundant lipid constituents are TAGs, free cholesterol, CEs, and phospholipids (especially PCs and PEs). Lipoproteins are the main player in exogenous, endogenous, and reverse cholesterol transport pathways, thus contributing predominantly to neutral lipids and phospholipids metabolism (Boren et al., 2022). In contrast, sphingolipid metabolism is more independent of lipoproteins (Borodzicz et al., 2015). Our findings provide a detailed overview of the differential effects of lipoproteins across a wide range of lipid classes and species and underscore the importance of accounting for their potentially confounding effects in lipidomics analyses.

The main strength of our study is that we were able to delineate the effects of a wide range of well‐characterized plasma lipids on two novel epigenetic estimators of biological aging. Moreover, we provided in‐depth analyses of the associations between the wide variation in the structure and fatty acid composition of a large array of lipid molecules on these biological aging estimators. Third, leveraging extensive individual‐level methylation array data, we were able to scrutinize the underlying biological pathways likely to be involved in mediating the effects of complex lipids on biological aging. In addition, our estimates are based on a broad age spectrum, ranging from 30 to 95 years, and are therefore likely to represent the lipidomic correlates of epigenetic aging across most of the adult lifespan. However, the cross‐sectional nature of our study precludes formal evaluation of the directionality of the effects. Based on findings from previous studies we nevertheless consider it likely that changes in lipid composition can alter DNA methylation (Dekkers et al., 2016; Jhun et al., 2021). Mendelian randomization analyses could help infer causality. Furthermore, longitudinal studies are needed to scrutinize the temporal dynamics of the relationship between lipids and biological aging. Moreover, we did not quantify some other potentially important lipid species, including phosphatidic acids, ether‐lipids of PCs, PSs, and acylcarnitines, which may also have an impact on biological aging.

## 4 CONCLUSION

In conclusion, diverse complex lipid species are associated with different rates of biological aging, with lipid class as well as fatty acid chain length and saturation as key determinants of their influence on biological aging. These findings emphasize the importance of investigating in‐depth lipidomics in aging research beyond the standard clinical lipid panel. Since lower levels of LPC species were predominantly associated with slower biological aging and have been linked to age‐related biological mechanisms (e.g., oxidative stress and mitochondrial dysfunction), they represent promising candidate human blood‐derived biomarkers of human aging. Finally, investigating the sources of different lipids which have disparate association patterns with biological aging may increase our understanding of the underlying biological mechanisms.

## 5 EXPERIMENTAL PROCEDURES

### 5.1 Study population

This study was based on the Rhineland Study, an ongoing single‐center, population‐based cohort study among people aged 30 years and above in Bonn, Germany. All individuals living in two pre‐defined recruitment areas are invited to participate in the study. The only exclusion criterion is an insufficient command of the German language to provide informed consent. One of the Rhineland Study's primary objectives is to identify determinants and markers of healthy aging, applying a deep‐phenotyping approach. At baseline, participants complete an 8‐h in‐depth multi‐domain phenotypic assessment, and various types of biomaterials are collected. Approval to undertake the study was obtained from the ethics committee of the University of Bonn, Medical Faculty. We obtained written informed consent from all participants in accordance with the Declaration of Helsinki.

For the current analysis, we used baseline data of the first 4471 participants of the Rhineland Study for whom complex lipids data were available. We excluded participants without methylation data (*n* = 290). The final analytical sample consisted of 4181 participants.

### 5.2 DNA methylation quantification

Genomic DNA was extracted from buffy coat fractions of anti‐coagulated blood samples using Chemagic DNA buffy coat kit (PerkinElmer, Germany) with Chemagic Magnetic Separation Module 1 and Chemagic Prime 8 Automated Workstation, and was subsequently bisulfite converted using the EZ‐96DNA Methylation‐Lightning™ MagPrep from Zymo according to the manufacturer's instructions. DNA methylation levels were measured on Illumina iScan using Illumina's Human MethylationEPIC BeadChip. The methylation level for each probe was derived as a beta value representing the fractional level of DNA methylation at that probe. Sample‐level and probe‐level quality control were performed using the “minfi” package (Fortin et al., 2017) in R. Probes with a missing rate > 1% (at a detection *p*‐value > 0.01) were excluded. Samples with sex mismatch or a missing rate at >1% across all probes were also excluded following previously published recommendation guidelines for analyzing methylation data (Wu & Kuan, 2018).

### 5.3 Estimation of biological age

Biological age was estimated as PhenoAge and GrimAge, which were calculated based on the algorithms developed by Levine et al. (2018) and Lu et al. (2019), using 513 and 1030 CpG sites, respectively. Discrepancies between an individual's chronological and estimated biological age (PhenoAge/GrimAge), referred to as AgeAccelPheno and AgeAccelGrim, were defined as the residual (in years) that results from regressing PhenoAge/GrimAge on chronological age. AgeAccelPheno and AgeAccelGrim represent the residual variation in estimated biological age independent of chronological age, indicating whether individuals have biologically aged faster or slower in comparison to their chronological age (Fox et al., 2023). Specifically, an increase in AgeAccelPheno and/or AgeAccelGrim with changing lipid levels is referred to as accelerated biological aging, whereas a decrease in AgeAccelPheno and/or AgeAccelGrim with changing lipid levels is referred to as slower biological aging (Jain et al., 2022; McCrory et al., 2021).

### 5.4 Complex lipid panel

Blood samples were collected between 7:00 and 9:45 AM from an antecubital or dorsal hand vein after overnight fasting. Plasma samples were shipped to Metabolon, USA. The shipment was done on dry ice in 96 format SBS racks and we used 0.7 mL Jacket External Screw Cap tubes of FluidX Tubes. All shipped samples had a volume of 500 μL. The absolute concentration (μM) of 14 lipid classes and the molecular species, namely cholesteryl esters (CE), monoacylglycerols (MAG), diacylglycerols (DAG), TAGs, phosphatidylcholines (PC), phosphatidylethanolamines (PE), phosphatidylinositols (PI), lysophosphatidylcholines (LPC), lysophosphatidylethanolamines (LPE), sphingomyelins (SM), ceramides (CER), hexosylceramides (HCER), lactosylceramides (LCER), and dihydroceramides (DCER), were measured in plasma samples (100 μL) using the True Mass Complex Lipid Panel (Metabolon, Research Triangle Park, NC, USA). The molecular species were defined by the number of carbons and the number of double bonds in up to two of the side chains [e.g., CE (16:1), DAG (14:0/18:1), and PC (16:0/22:6)]. Besides individual molecular species, fatty acid composite measures were also calculated. These composite measures contained the absolute concentration of all lipid species of a specific length and saturation, for example, DAG [FA14:1] summarizes all DAGs with at least one fatty acid tail of 14 carbons and 1 double bond.

An automated Butanol‐Methanol (BUME) method was used for lipid extraction (van Dijk et al., 2012). After extracts were dried and reconstituted, they were transferred to vials for infusion mass‐spectroscopy analysis. The samples were analyzed using mass spectroscopy via both positive and negative mode electrospray on Shimadzu LC with nano PEEK tubing and the Sciex SelexIon‐5500 QTRAP. Briefly, The Complex Lipid Panel uses flow injection analysis (FIA), differential mobility separation (DMS), and multiple reaction monitoring (MRM) technologies (FIA‐DMS‐MRM) in the lipidomic analysis. The lipid sample solution is continuously infused into the mass spectrometer without the use of any chromatographic column. After ionization in the source of the mass spectrometer, lipids are introduced into the SelexION DMS cell, which acts as a lipid filter that permits a specific lipid class to pass into the mass spectrometer. Then, the selected lipid class enters the mass spectrometer for MRM analysis, in which the first quadrupole (Q1) filters on the basis of the m/z of the intact lipid species, while the third quadrupole (Q3) filters on the basis of the m/z of a characteristic fragment of that same lipid species, such as one of the fatty acid side chains. The Complex Lipid Panel included more than 50 stable isotope internal standards (Table S1), which were added before sample extraction to ensure accurate quantification of lipids across and within classes. These internal standards cover all of the lipid classes measured except for phosphatidylinositols (PIs), and PE internal standards were used as proxies for the PI class. In addition, a small aliquot of each sample was pooled to create a CMTRX technical replicate sample, which was then injected periodically throughout the platform run to ensure that no significant batch‐to‐batch variation occurs within a sample set. Moreover, samples were randomized across the platform run with CMTRX samples spaced evenly among the injections. As the samples were run across multiple days, the data were normalized against their run day and inter‐batch median using the values from CMTRX samples, and then multiplied by the median background subtraction value, as obtained from filtered water, to regain the absolute concentrations. By multiplying the concentration of the internal standard with the ratio between the signal intensity of the target compound and the internal standard, the absolute concentration of lipid species was quantified and subdivided into neutral, phospho‐, and sphingolipids based on their fundamental biochemical structure (Fahy et al., 2011). Lipid class concentrations were calculated by summing up all molecular species within a class, and fatty acid compositions were calculated by summing individual species with specific biochemical characteristics within each class. The coefficients of variation (CVs) of lipid concentrations were all below 10%. The median CV of species at 1 μM concentration in plasma samples was approximately 5%.

In total, 1050 molecular species and 278 fatty acid compositions covering these 14 classes were measured. Individual lipid species that contained more than 90% missing values across all the participants were not included (86 molecular species and 11 fatty acid compositions), leaving a total of 964 molecular species and 267 fatty acid compositions for the analyses (Table S2). Lipids with zero double bonds are defined as saturated fatty acids (SFA), lipids with one double bond are defined as monounsaturated fatty acids (MUFA), and lipids with more than one double bond are defined as polyunsaturated fatty acids (PUFA).

### 5.5 Demographic and health variables

We included age and sex as demographic factors. Smoking status was defined as “current smoker” or “non‐current smoker” based on self‐report. Missing smoking values were imputed based on cotinine metabolite levels: individuals with a cotinine level exceeding the non‐current smoker sample‐defined 97.5 percentile were classified as smokers (St Helen et al., 2012). Body mass index (BMI) was calculated as weight in kilograms dived by height in meters squared. Systolic blood pressure (SBP) and diastolic blood pressure (DBP) were measured three times (separated by 10 min intervals), using an oscillometric blood pressure device (Omron 705 IT). The measurements were performed while people were sitting in a resting chair in a quiet environment, and the average of the second and third measurements was used in the analyses. Participants were considered to have diabetes if they had a self‐reported physician diagnosis of diabetes, glycated hemoglobin (%) levels of 6.5% or more, or used anti‐diabetic medication. Hypertension was defined as a self‐reported physician diagnosis of hypertension, an average systolic blood pressure ≥ 140 mmHg and/or diastolic blood pressure ≥ 90 mmHg, or the use of antihypertensive drugs. LDL, HDL, and total cholesterol and triglyceride concentrations (mg/dL) were measured using standard methods at the local clinical chemistry laboratory of the University Hospital of Bonn.

### 5.6 Statistical analyses

Statistical analyses were performed in R (version 4.3.0, The R Foundation). The attached base packages include “stats,” “graphics,” “grDevices,”, “utils,” “datasets,” “methods,” and “base.” Data were summarized as mean ± standard deviation (SD) or counts with proportions, for continuous and categorical variables, respectively. Differences between women and men were compared using linear regression for continuous variables, and logistic regression for categorical variables adjusting for age. Age and sex‐adjusted partial correlations were used to assess the correlations among standard clinical lipid measures (i.e., LDL, HDL, total cholesterol, TAG, LDL/HDL ratio), BMI, and the 14 main lipid classes and the partial‐correlation matrix was generated using “corrplot” R package. All lipid variables were z‐transformed to have a mean of 0 and a standard deviation of 1 before further analyses to enable a better comparison of the effect sizes across different lipid classes. We used complete data without the imputation of missing values for all analyses.

#### 5.6.1 Association analysis

We used multiple linear regression analyses to quantify the association between each lipid class, molecular species, fatty acid composition (independent variables), and AgeAccelPheno/AgeAccelGrim (dependent variables). The base model was adjusted for sex, batch information of lipids and methylation, and smoking status. As HDL and LDL transport lipids in the circulation which could confound the association between complex lipids and biological age estimators, we further adjusted for HDL and LDL, both separately and jointly. To assess whether the associations of lipid species with AgeAccelPheno/AgeAccelGrim were independent of BMI, we additionally adjusted for BMI. We used the false discovery rate (FDR) method to account for multiple comparisons, considering FDR <0.05 as statistically significant.

The overall patterns between all lipid classes/species and AgeAccelPheno/AgeAccelGrim were shown as forest plots. Patterns across all species of specific lipid classes were also shown as forest plots. To assess whether the effect (strength and direction) of the associations depended on the total number of carbons and double bonds in the lipids, the effect estimates of the lipid species were plotted as circles with their position in the 2‐dimensional lipid class graphs determined by the total carbon number (*x*‐axis) and double bonds (*y‐*axis). To assess whether the effect (strength and direction) of the associations depended on the number of carbons in one specific fatty acid tail, heat maps were created for the effect estimates of the lipid species at FDR <0.05 level. To examine whether the associations differed by the degree of saturation in each lipid molecular species, beta estimates across lipid classes were shown as forest plots. The plots were generated using the “ggplot2,” “ggpubr,” “ggrepel,” and “gridExtra” R packages.

We also assessed the association between chronological age and the levels of the 14 lipid classes in seven different age groups (i.e., 30–39 years old, 40–49 years old, 50–59 years old, 60–69 years old, 70–79 years old, 80–89 years old, and ≥90 years old), adjusting for sex.

#### 5.6.2 Sex interaction and sex‐stratified analysis

To examine sex differences between lipid class and lipid molecular species and AgeAccelPheno/AgeAccelGrim, we assessed the interaction effects between sex and each lipid species on AgeAccelPheno/AgeAccelGrim. In case statically significant sex‐lipid interactions were identified, additional sex‐stratified analyses were performed.

#### 5.6.3 Pathway analysis

Epigenome‐wide association analyses were performed to examine the association between AgeAccelPheno/AgeAccelGrim‐associated lipid species (independent variable) and DNA methylation level (outcome) using multiple linear regression while adjusting for age, sex, smoking status, batch information of lipids and methylation, first 10 genetic principal components (PCs) which account for residual population stratification. The associated CpGs were used as proxies for each lipid to interrogate the Kyoto Encyclopedia of Genes and Genomes (KEGG) database for pathway analysis using g:Profiler (https://biit.cs.ut.ee/gprofiler/). A bar plot to visualize the KEGG pathway results was generated using “ggplot2” R package.

## AUTHOR CONTRIBUTIONS

**Dan Liu** was involved in conceptualization, methodology, formal analysis, writing—original draft preparation, and visualization; **N. Ahmad Aziz** was involved in conceptualization, methodology, supervision, and writing—reviewing and editing**; Elvire N. Landstra** was involved in methodology and writing—reviewing and editing; **Monique M.B. Breteler** was involved in conceptualization, methodology, resources, writing—reviewing and editing, supervision, data curation, and funding acquisition.

## CONFLICT OF INTEREST STATEMENT

None of the authors has any conflict of interest.

## Supporting information


Data S1.

## Data Availability

The Rhineland Study's dataset is not publicly available because of data protection regulations. Access to data can be provided to scientists in accordance with the Rhineland Study's Data Use and Access Policy. Requests for further information or to access the Rhineland Study's dataset should be directed to rs‐duac@dzne.de. The corresponding author takes responsibility for the integrity of the data.
