# Supplementary material for: The lipidomic correlates of epigenetic aging across the adult lifespan: A population‐based study
Source: Aging Cell. 2023 Jul 26;22(9):e13934. doi: 10.1111/acel.13934 (PMC10497837; doi:10.1111/acel.13934)
Supplement: Supplementary file 1 — Data S1. [file ACEL-22-e13934-s001.docx]

**The Lipidomic Correlates of Epigenetic Aging across the Adult Lifespan: A Population-based Study**

**Authors**

Dan Liu^1*^, N. Ahmad Aziz^1,2^, Elvire Nadieh Landstra^1^, Monique M.B. Breteler^1,3^

**Affiliations**

^1^Population Health Sciences, German Center for Neurodegenerative Diseases (DZNE), Bonn, Germany

^2^Department of Neurology, Faculty of Medicine, University of Bonn, Bonn, Germany

^3^Institute for Medical Biometry, Informatics and Epidemiology (IMBIE), Faculty of Medicine, University of Bonn, Bonn, Germany

**Supplementary Information**

**Supplementary Table 1.** **List of internal standards and their concentration used in the complex lipid panel**

**Supplementary** **Table 2. Lipid class and molecular species measured in complex lipids panel and were included in the analyses**

**Supplementary** **Figure 1. Age, sex, batch-adjusted partial-correlation matrix of standard clinical lipid measures, BMI, and main lipid classes**

**Supplementary** **Figure 2. The association between chorological age and 14 lipid class levels in different age groups**

**Supplementary** **Figure 3. Associations of total number of carbons and double bonds in neutral lipids and sphingolipids with AgeAccelPheno and AgeAccelGrim**

**Supplementary** **Figure 4. Interaction effects between sex and lipid species on AgeAccelPheno and AgeAccelGrim**

**Supplementary** **Figure 5. Sex difference between lipid species concentration and AgeAccelPheno**

**Supplementary** **Figure 6. Sex difference between lipid species concentration and AgeAccelGrim**

| **Supplementary Table 1.** **List of internal standards and their concentration used in the complex lipids panel** | | | |
| --- | --- | --- | --- |
| Lipid class | Internal Standards | MW (g.mol^-^1) | Concentration (mg.L^-^1) |
| CE | dCE(16:0) | 631,62 | 0,13 |
|  | dCE(16:1) | 629,61 | 0,13 |
|  | dCE(18:1) | 657,64 | 0,57 |
|  | dCE(18:2) | 655,62 | 1,43 |
|  | dCE(20:3) | 681,64 | 0,15 |
|  | dCE(20:4) | 679,62 | 0,18 |
|  | dCE(20:5) | 677,61 | 0,18 |
|  | dCE(22:6) | 703,62 | 0,22 |
| DAG | dDAG(16:0_16:0) | 577,56 | 0,004 |
|  | dDAG(16:0_18:0) | 605,59 | 0,005 |
|  | dDAG(16:0_18:1) | 603,57 | 0,006 |
|  | dDAG(16:0_18:2) | 601,56 | 0,005 |
|  | dDAG(16:0_18:3) | 599,54 | 0,00135 |
|  | dDAG(16:0_20:4) | 625,56 | 0,0015 |
|  | dDAG(16:0_20:5) | 623,54 | 0,00145 |
|  | dDAG(16:0_22:6) | 649,56 | 0,0016 |
| CER | dCER(d16:0) | 546,97 | 0,02 |
| DCER | dDCER(16:0) | 548,99 | 0,004 |
| HCER | dHCER(16:0) | 709,11 | 0,03 |
| LCER | dLCER(16:0) | 871,25 | 0,03 |
| SM | dSM(16:0) | 709,61 | 0,1 |
|  | dSM(18:1) | 735,62 | 0,1 |
|  | dSM(24:0) | 821,73 | 0,1 |
|  | dSM(24:1) | 819,72 | 0,1 |
| LPC | dLPC(16:0) | 504,69 | 0,1 |
| LPE | dLPE(18:0) | 486,64 | 0,05 |
| PC | dPC(16:0_16:1) | 740,6 | 0,0575 |
|  | dPC(16:0_18:1) | 768,63 | 0,2525 |
|  | dPC(16:0_18:2) | 766,62 | 0,255 |
|  | dPC(16:0_18:3) | 764,6 | 0,065 |
|  | dPC(16:0_20:3) | 792,63 | 0,0725 |
|  | dPC(16:0_20:4) | 790,62 | 0,2775 |
|  | dPC(16:0_20:5) | 788,6 | 0,07 |
|  | dPC(16:0_22:4) | 818,65 | 0,075 |
|  | dPC(16:0_22:5) | 816,63 | 0,0775 |
|  | dPC(16:0_22:6) | 814,62 | 0,145 |
| PE | dPE(18:0_18:1) | 750,59 | 0,01 |
|  | dPE(18:0_18:2) | 748,58 | 0,01 |
|  | dPE(18:0_18:3) | 746,56 | 0,0021 |
|  | dPE(18:0_20:3) | 774,59 | 0,0027 |
|  | dPE(18:0_20:4) | 772,58 | 0,01 |
|  | dPE(18:0_20:5) | 770,56 | 0,0022 |
|  | dPE(18:0_22:5) | 798,6 | 0,0024 |
|  | dPE(18:0_22:6) | 796,58 | 0,01 |
| TAG | dTAG50:1-FA16:0 | 841,81 | 0,13 |
|  | dTAG52:1-FA18:0 | 869,84 | 0,14 |
|  | dTAG52:2-FA18:1 | 867,82 | 0,14 |
|  | dTAG52:3-FA18:2 | 865,8 | 0,14 |
|  | dTAG52:4-FA18:3 | 863,79 | 0,04 |
|  | dTAG54:4-FA20:3 | 891,82 | 0,04 |
|  | dTAG54:5-FA20:4 | 889,8 | 0,04 |
|  | dTAG56:7-FA22:6 | 913,8 | 0,038 |

**Supplementary** **Table 2. Lipid class and molecular species measured in complex lipids panel and were included in the analysis**

| Group | Abbreviation | Lipid class | Molecular species | | Fatty acid composition | |
| --- | --- | --- | --- | --- | --- | --- |
|  |  |  | No. of measured  (n= 1050) | No. of included in the analysis  (n = 964) | No. of measured  (n = 278) | No. of included in the analysis  (n = 267) |
| Neutral lipids | CE | Cholesteryl esters | 27 | 27 | 26 | 26 |
|  | MAG | Monoacylglycerols | 27 | 27 | 26 | 26 |
|  | TAG | Triacylglycerols | 519 | 519 | 21 | 21 |
|  | DAG | Diacylglycerols | 59 | 59 | 19 | 19 |
| Phospholipids | PC | Phosphatidylcholines | 121 | 106 | 23 | 23 |
|  | PE | Phosphatidylethanolamines | 80 | 42 | 21 | 16 |
|  | PE(O) | phosphatidylethanolamine ether | 26 | 17 | - | - |
|  | PE(P) | phosphatidylethanolamine plasmalogen | 53 | 36 | - | - |
|  | PI | Phosphatidylinositols | 29 | 28 | 13 | 13 |
|  | LPC | Lysophosphatidylcholines | 22 | 19 | 21 | 18 |
|  | LPE | Lysophosphatidylethanolamines | 21 | 18 | 20 | 17 |
| Sphingolipids | SM | Sphingomyelins | 13 | 13 | 12 | 12 |
|  | CER | Ceramides | 13 | 13 | 12 | 12 |
|  | HCER | Hexosylceramides | 13 | 13 | 12 | 12 |
|  | LCER | Lactosylceramides | 13 | 13 | 12 | 12 |
|  | DCER | Dihydroceramides | 14 | 14 | 12 | 12 |
|  | TOTAL FA | Total fatty acid compositions | - | - | 28 | 28 |

**Supplementary** **Figure 1. Age, sex, batch-adjusted partial-correlation matrix of standard clinical lipid measures, BMI, and main lipid classes**

**
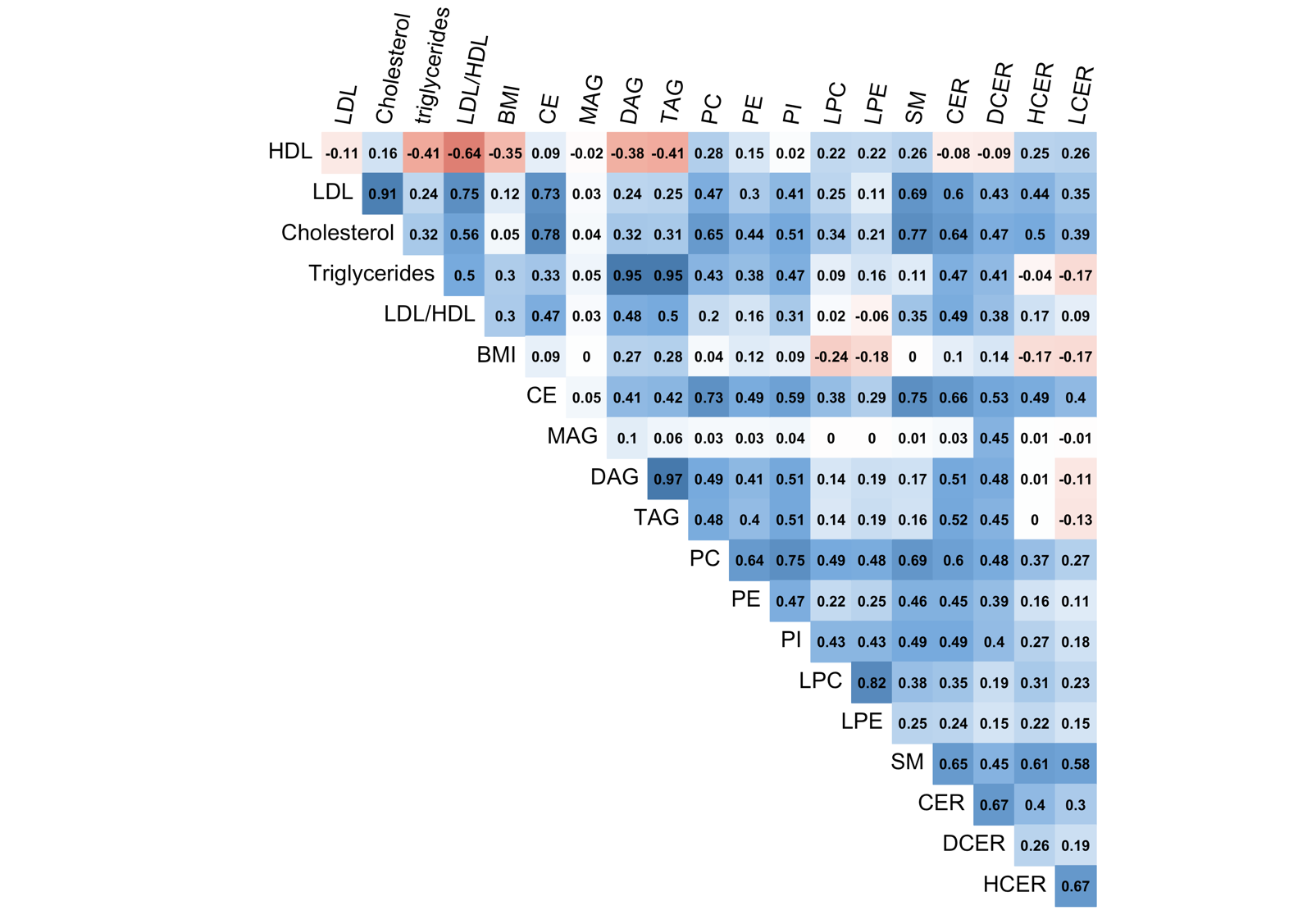
**

Abbreviations: high-density-lipoprotein (HDL), low-density-lipoprotein (LDL), body mass index (BMI), cholesteryl esters (CE), monoacylglycerols (MAG), diacylglycerols (DAG), triacylglycerols (TAG), phosphatidylcholines (PC), phosphatidylethanolamines (PE), phosphatidylinositols (PI), lysophosphatidylcholines (LPC), lysophosphatidylethanolamines (LPE), sphingomyelins (SM), ceramides (CER), dihydroceramides (DCER), hexosylceramides (HCER), lactosylceramides (LCER)

**Supplementary** **Figure 2. The association between chorological age and 14 lipid class levels in different age groups**

**
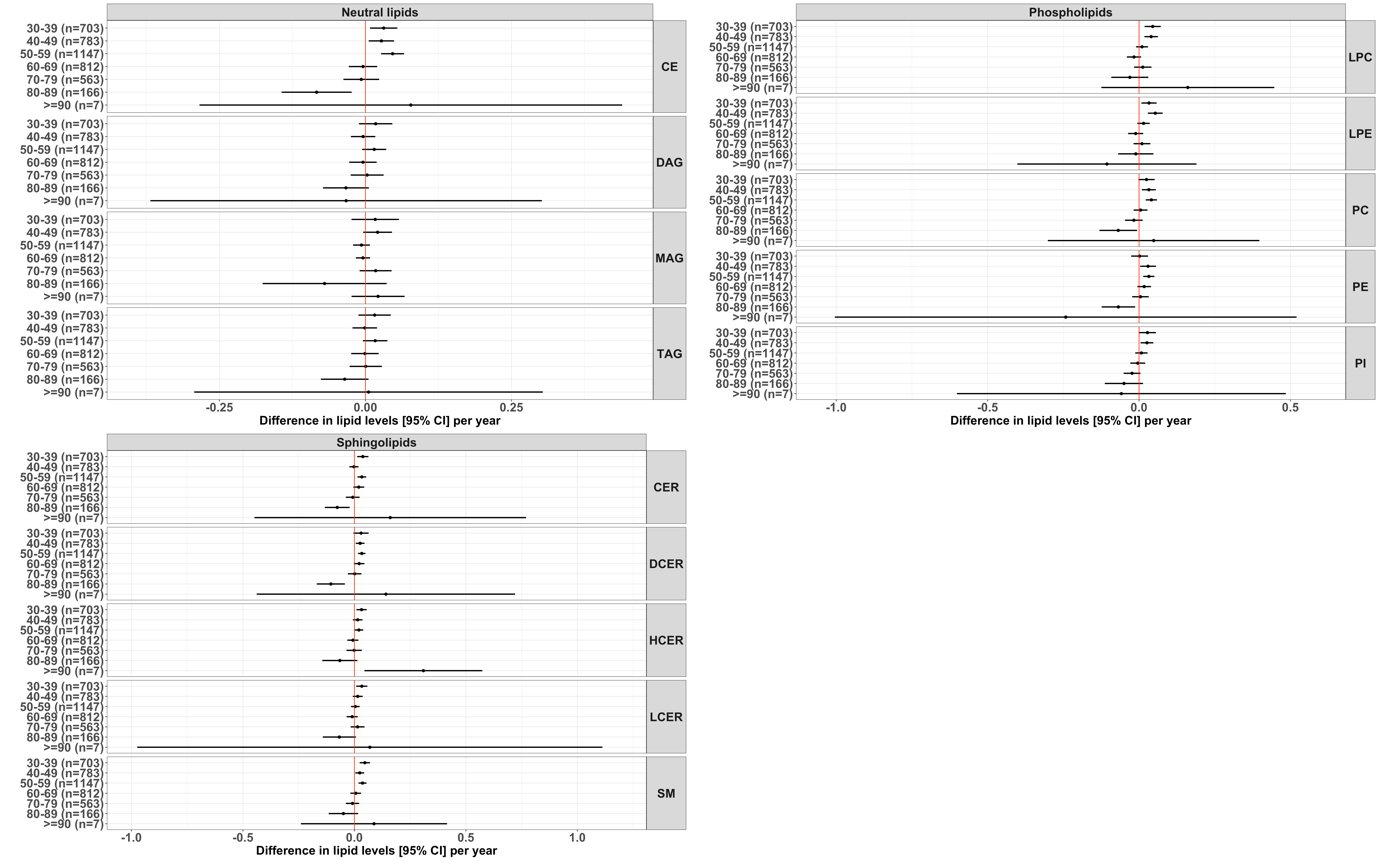
**

Numbers between brackets behind each group indicate the number of participants in the corresponding age group.

Abbreviations: cholesteryl esters (CE), monoacylglycerols (MAG), diacylglycerols (DAG), triacylglycerols (TAG), phosphatidylcholines (PC), phosphatidylethanolamines (PE), phosphatidylinositols (PI), lysophosphatidylcholines (LPC), lysophosphatidylethanolamines (LPE), sphingomyelins (SM), ceramides (CER), dihydroceramides (DCER), hexosylceramides (HCER), lactosylceramides (LCER)

**Supplementary** **Figure 3. Associations of total number of carbons and double bonds in neutral lipids and sphingolipids with AgeAccelPheno and AgeAccelGrim**

**
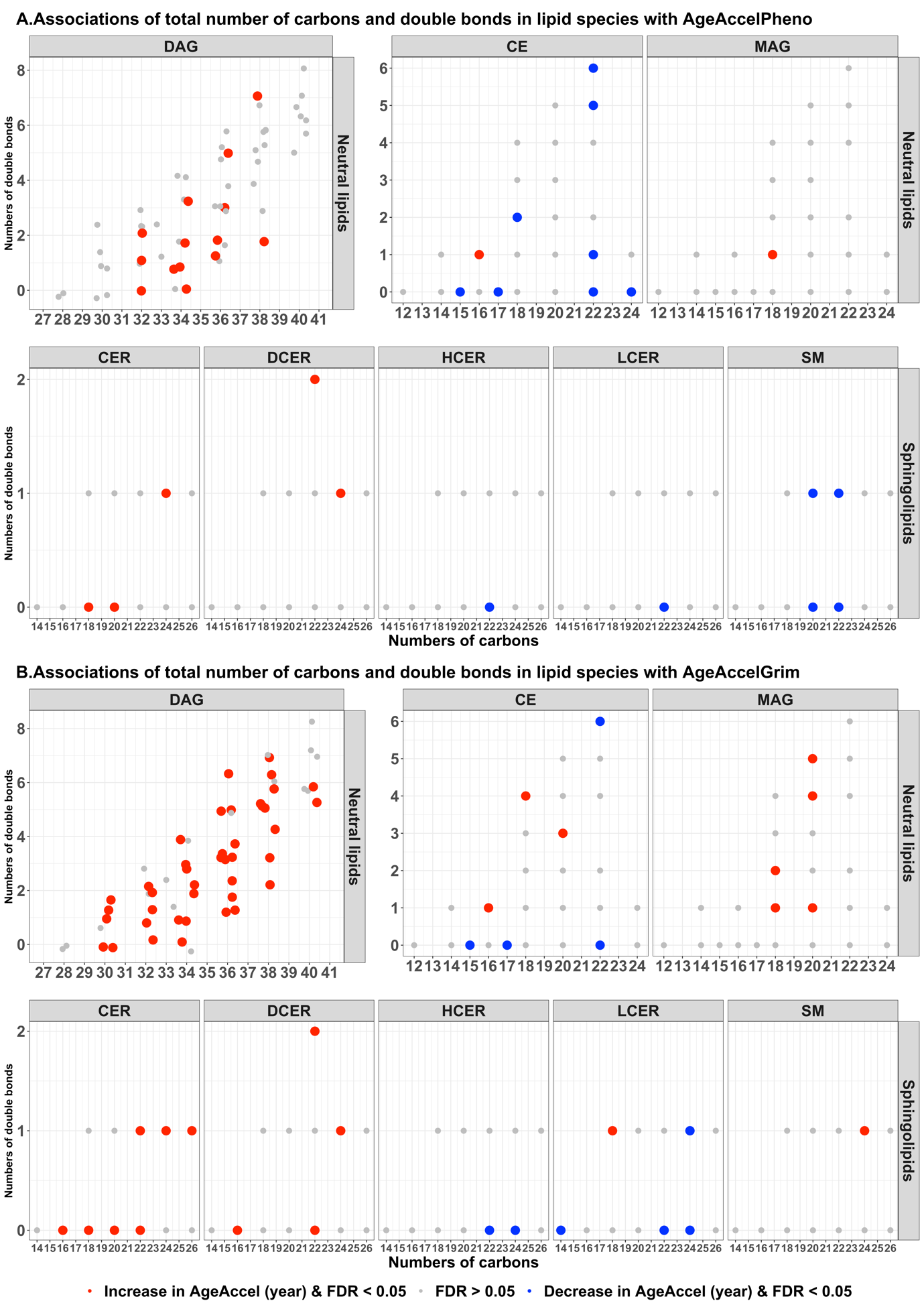
**

Individual lipid species are depicted by filled circles and arranged by lipid class according to the number of total carbon atoms (x-axes) and number of double bonds (y-axes). Color indicates the magnitude of effect size, and circle size corresponds to the significance level. Lipids with the same number of carbon atoms and double bonds are pulled apart vertically to increase their visibility. Abbreviations: diacylglycerols (DAG), cholesteryl esters (CE), monoacylglycerols (MAG), ceramides (CER), dihydroceramides (DCER), hexosylceramides (HCER), lactosylceramides (LCER), sphingomyelins (SM), false discovery rate (FDR).

**Supplementary** **Figure 4. Interaction effects between sex and lipid species on AgeAccelPheno and AgeAccelGrim**

**
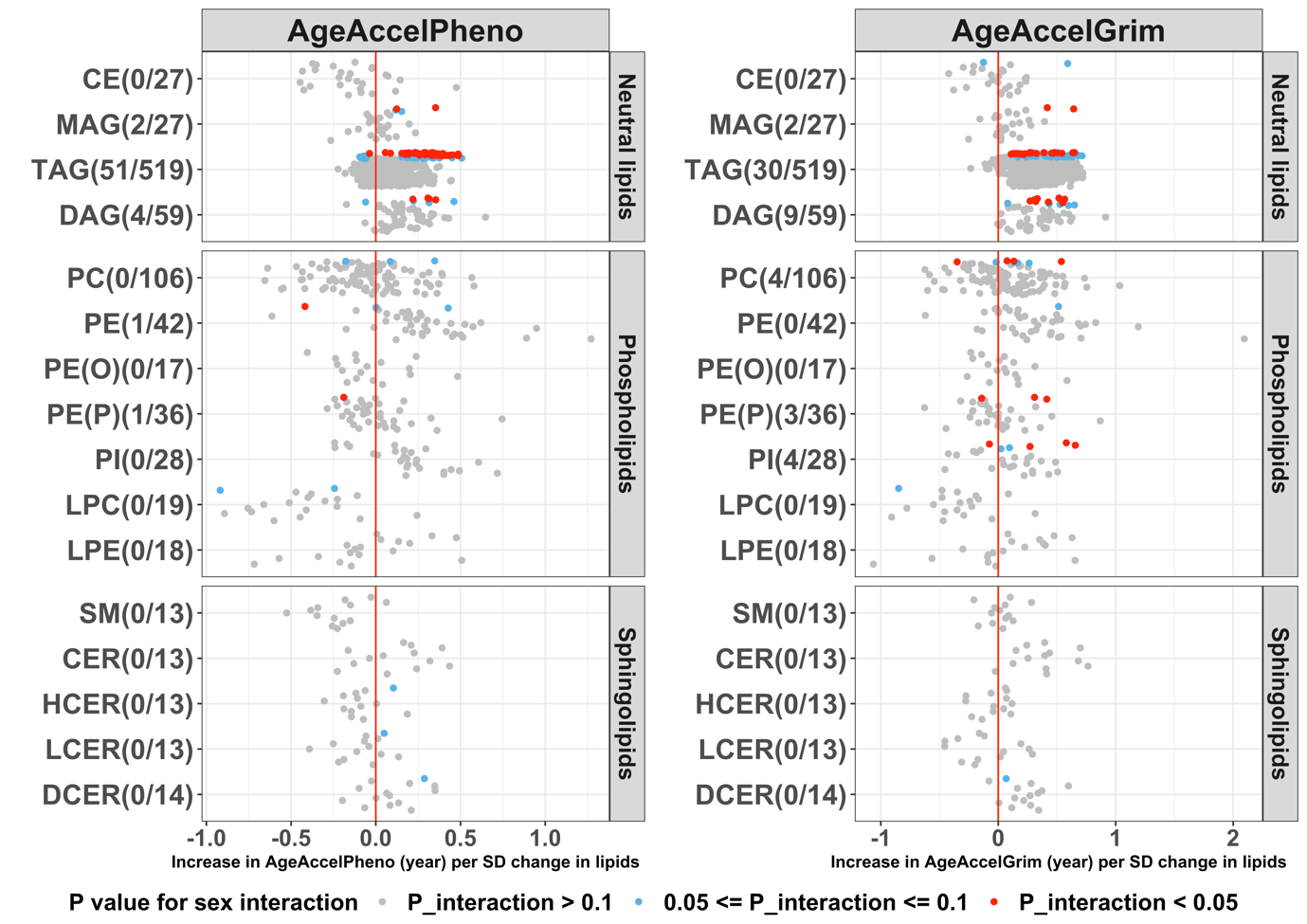
**

Numbers between brackets behind each class indicate number of significant sex-interaction lipid species/total number of lipid species in that class.

Abbreviations: cholesteryl esters (CE), monoacylglycerols (MAG), triacylglycerols (TAG), diacylglycerols (DAG), phosphatidylcholines (PC), phosphatidylethanolamines (PE), phosphatidylethanolamine ether (PE(O)), phosphatidyl-ethanolamine plasmalogen (PE(P)), phosphatidylinositols (PI), lysophosphatidylcholines (LPC), lysophosphatidylethanolamines (LPE), sphingomyelins (SM), ceramides (CER), hexosylceramides (HCER), lactosylceramides (LCER), dihydroceramides (DCER).

**Supplementary** **Figure 5. Sex difference between lipid species concentration and AgeAccelPheno**

**
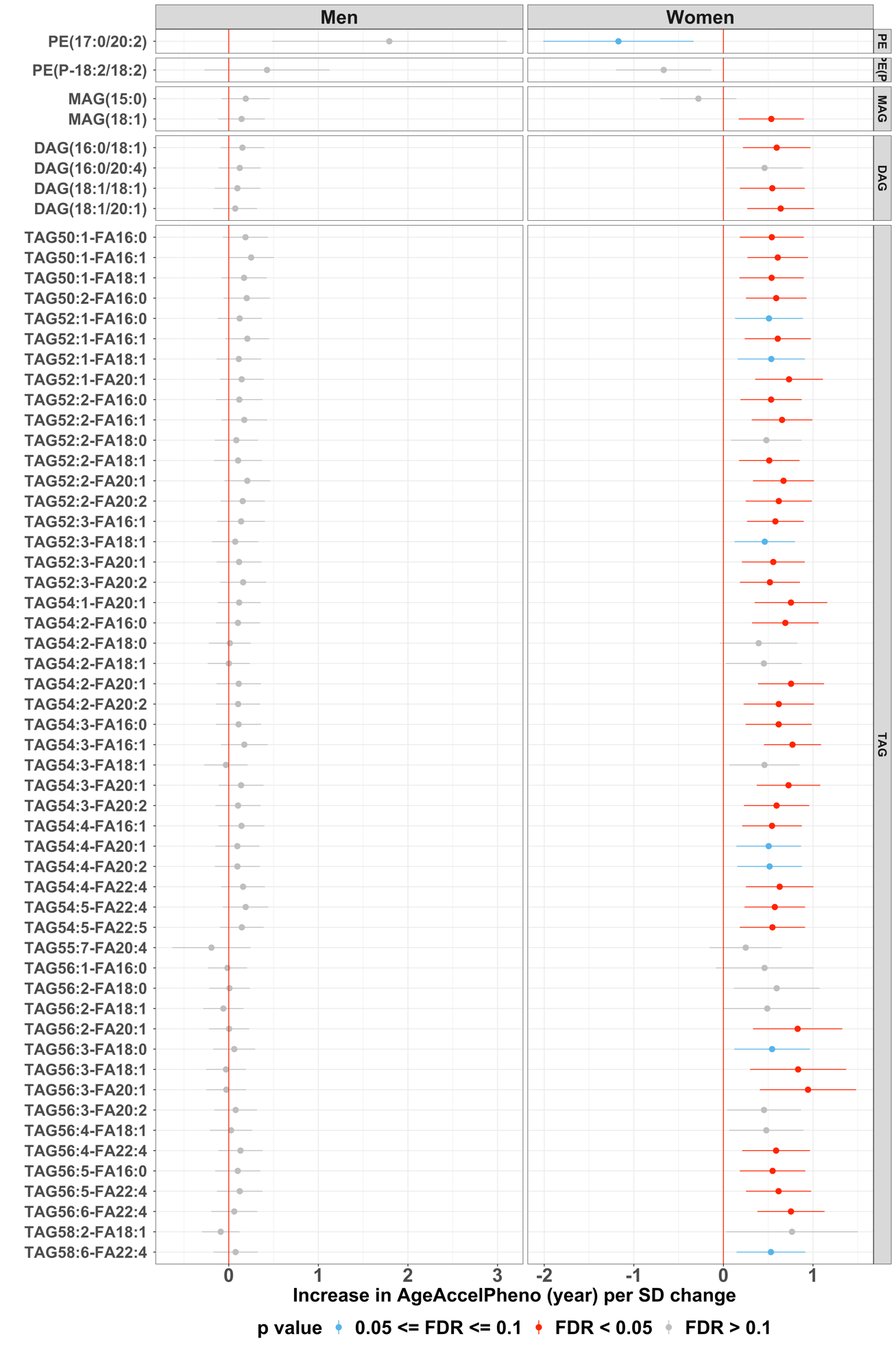
**

Abbreviations: phosphatidylethanolamines (PE), monoacylglycerols (MAG), diacylglycerols (DAG), triacylglycerols (TAG).

**Supplementary** **Figure 6. Sex difference between lipid species concentration and AgeAccelGrim**

**
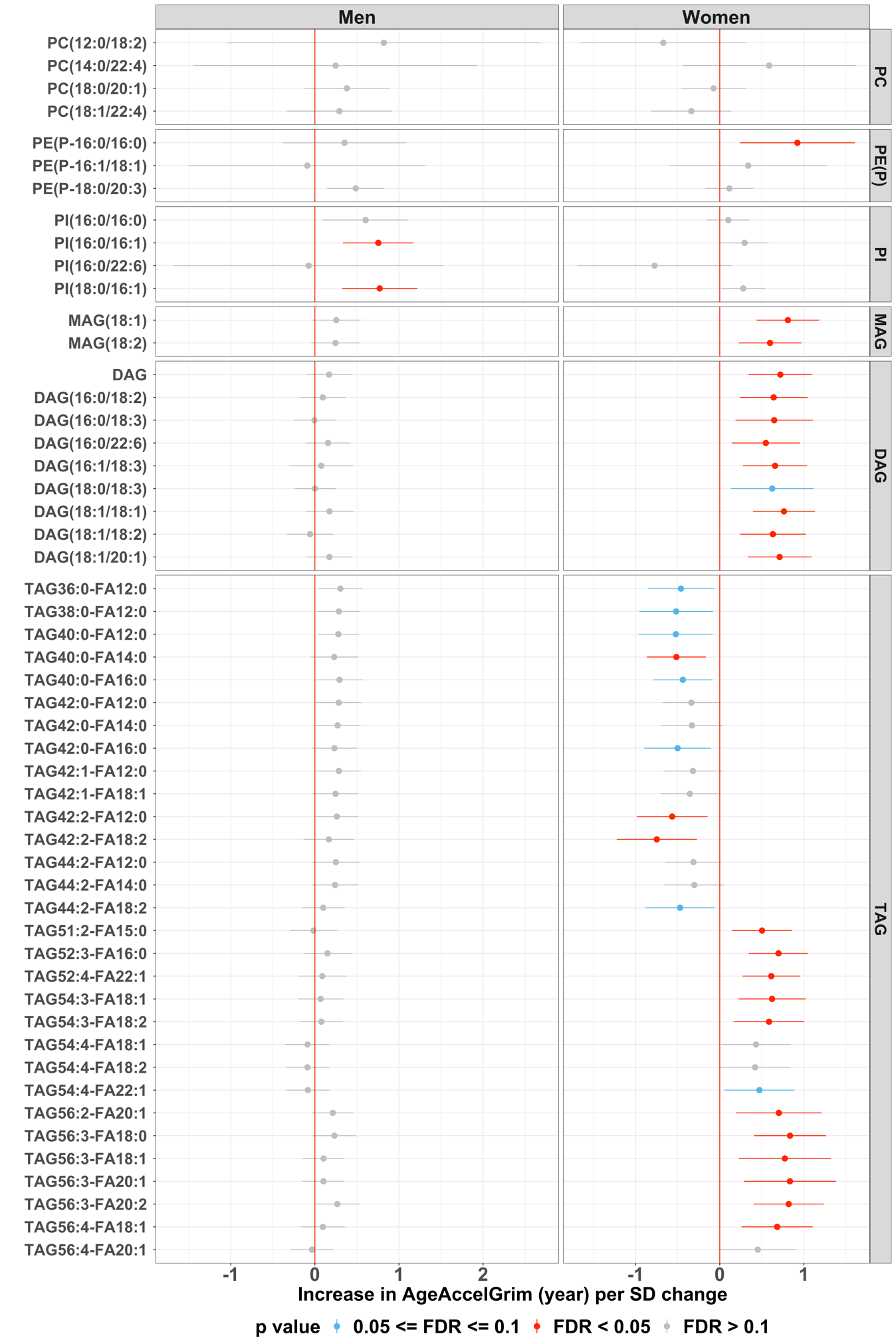
**

Abbreviations: phosphatidylcholines (PC), phosphatidylethanolamines (PE), phosphatidylinositols (PI), monoacylglycerols (MAG), diacylglycerols (DAG), triacylglycerols (TAG) .
